# Development and validation of a nomogram predicting osteoporosis risk in rheumatoid arthritis

**DOI:** 10.3389/fmed.2026.1747090

**Published:** 2026-04-29

**Authors:** Lujing Wang, Yueting Gu, Xiaochun Zhang, Xinglan Bao, Yan Chen, Hongjian Yuan

**Affiliations:** 1 Department of Rheumatology, Taizhou Second People’s Hospital Affiliated to Yangzhou University, Taizhou, Jiangsu, China; 2 Qingpu Branch of Zhongshan Hospital Affiliated to Fudan University, Shanghai, China; 3 Department of Colorectal Surgery, The Second Affiliated Hospital of Nanjing University of Chinese Medicine, Nanjing, Jiangsu, China

**Keywords:** alkaline phosphatase, ApoA1, free fatty acids, nomogram, osteoporosis, rheumatoid arthritis

## Abstract

**Background:**

Rheumatoid arthritis (RA) increases the risk of osteoporosis, but tools that integrate RA-specific clinical and metabolic factors to predict osteoporosis risk are limited. We aimed to develop and validate a practical risk prediction nomogram for osteoporosis in RA patients.

**Methods:**

In this single-center retrospective study, 349 RA patients with available DXA data were analyzed; 132 (37.8%) had osteoporosis. A training cohort (*n* = 250; osteoporosis = 92) and a temporal validation cohort (*n* = 99; osteoporosis = 40, enrolled later in the study period) were used. Candidate predictors included clinical, functional, and laboratory variables. Stepwise backward logistic regression identified independent predictors that were incorporated into a nomogram. Model performance was assessed by discrimination (AUROC), calibration (calibration curve and Hosmer–Lemeshow test), decision curve analysis (DCA), and risk stratification.

**Results:**

Female sex, higher health assessment questionnaire-disability index (HAQ-DI), elevated alkaline phosphatase (ALP), increased ApoA1/ApoB ratio, higher free fatty acids (FFA), and lower body mass index (BMI) were independent predictors of osteoporosis and were included in the nomogram. The model yielded AUROCs of 0.812 (training) and 0.788 (validation), showed good calibration (Hosmer–Lemeshow *p* > 0.05), and provided positive net benefit across a range of threshold probabilities in DCA. Nomogram-based risk strata (low/medium/high) discriminated osteoporosis risk with statistically significant odds ratios for medium and high groups.

**Conclusion:**

The proposed nomogram, built from readily available clinical and laboratory measures, demonstrates good discrimination, calibration, and clinical utility for identifying RA patients at elevated risk of osteoporosis, and may facilitate targeted screening and early intervention. However, the model’s performance in diverse populations remains unknown, and prospective multicenter external validation is essential before any clinical application.

## 1 Introduction

Rheumatoid arthritis (RA) is a systemic autoimmune disease characterized primarily by chronic inflammatory arthritis, which leads to cartilage and bone destruction. It is marked by synovitis, joint damage, bone loss, and a range of systemic complications (1). Patients with RA are exposed to a persistent inflammatory milieu and often experience limited mobility, poor nutritional status, and prolonged use of corticosteroids or glucocorticoids, all of which contribute to impaired bone metabolism. As a result, they exhibit decreased bone mineral density (BMD) and a significantly higher prevalence of osteoporosis compared with the general population (2). Previous studies have reported that the risk of fracture in RA patients is more than twice that observed in individuals without RA (3). According to recent epidemiological data, the overall prevalence of osteoporosis among RA patients is approximately 27.6% (95% CI: 23.9–31.3%), highlighting a clinical concern that warrants careful management and prevention of complications (4). Osteoporosis not only diminishes quality of life but also increases the risk of fragility fractures, disability, and mortality. Therefore, early screening and intervention for bone mineral density loss are of great importance in the comprehensive management of RA patients.

Currently, the gold standard for diagnosing osteoporosis is the measurement of BMD (5). Osteoporosis is a systemic skeletal disorder characterized by bone loss and microarchitectural deterioration of bone tissue, leading to increased bone fragility and susceptibility to fractures, particularly in the hip, spine, and wrist. Therefore, identifying high-risk populations for osteoporosis through early, comprehensive, and accurate screening is of paramount importance. Local bone erosion and systemic osteoporosis share similar pathological mechanisms, both closely linked to inflammation and disease activity, which further exacerbate generalized bone loss. As local bone erosion progresses, patients with RA exhibit a progressive decline in BMD and a higher incidence of osteoporosis. Although clinical tools such as the FRAX^®^ score are widely used to assess osteoporosis risk, their predictive power in the RA population remains limited, as they do not fully account for RA-specific pathophysiological features, including disease activity, functional impairment, and distinct inflammatory and metabolic biomarkers (6).

Recognizing the need for practical screening tools, Lems and Dijkmans proposed preliminary clinical criteria (age, disease activity, immobility) to guide bone densitometry in RA patients (7). Nolla et al. subsequently evaluated these criteria in 128 postmenopausal women with RA, reporting a sensitivity of 86% and specificity of 43% for identifying osteoporosis, suggesting utility as a screening method despite modest specificity (8). Kvein et al. later developed a data-driven logistic regression algorithm in female RA patients, achieving sensitivities of 50–60% and specificities of 80–90%, though they concluded that clinical markers alone could not provide sufficient accuracy for reliable clinical use (9). A number of RA-focused efforts have sought to improve prediction in this population. Chen et al. applied machine learning to predict fracture risk in elderly-onset RA, achieving AUCs of 0.713–0.872 (10). Lee et al. identified traditional risk factors (age, low BMI, disease duration, glucocorticoid use, HAQ score) and additionally uncovered socioeconomic predictors such as income and education (11). Yan et al. developed a logistic regression model incorporating age, disease duration, DAS28, anti-CCP, and ultrasound-detected bone erosion, reporting excellent discrimination (C-index > 0.94) (12). Other studies have explored novel biomarkers, including the neutrophil percentage-to-albumin ratio and the second metacarpal cortical index (13). Emerging evidence also implicates lipid metabolism, with HDL cholesterol independently associated with osteoporosis in RA (14). Despite these advances, existing models have notable limitations. Many rely on single biomarkers or conventional variables and fail to integrate the complex interplay among inflammation, metabolic dysfunction, and bone metabolism central to RA pathophysiology. Others incorporate imaging parameters or novel biomarkers not routinely available, limiting clinical applicability.

In recent years, increasing attention has been paid to the role of lipid metabolism markers, such as apolipoproteins and free fatty acids (FFAs), in bone metabolism, suggesting that metabolic pathways may contribute to the development of osteoporosis. Apolipoprotein A1 (ApoA1), the major component of high-density lipoprotein (HDL), exerts anti-inflammatory and antioxidant effects, whereas apolipoprotein B (ApoB), predominantly found in low-density lipoprotein (LDL), is associated with pro-inflammatory and atherogenic processes. Thus, the ApoA1/ApoB ratio may serve as a comprehensive indicator reflecting the balance between systemic inflammation and lipid metabolism (15). A decreased ratio may indicate a more pronounced pro-inflammatory and pro-oxidative state, detrimental to bone health. Moreover, elevated levels of FFAs have been shown to induce oxidative stress and the release of inflammatory cytokines, potentially stimulating osteoclast activity directly or indirectly and thereby promoting bone resorption (16).

Given the above background, integrating multiple measurable indicators into a comprehensive risk prediction model—particularly through statistical modeling to assess the weight and contribution of different factors and validating the model within a defined cohort—holds significant clinical value for the early identification of high-risk RA patients and the development of individualized prevention strategies. Based on this rationale, we developed a binary logistic regression model incorporating ApoA1/ApoB ratio, BMI, FFA, Health Assessment Questionnaire-Disability Index (HAQ-DI), alkaline phosphatase (ALP), and sex as predictive variables. Using these parameters, we constructed a nomogram to estimate the risk of osteoporosis in RA patients and further validated its predictive performance through independent sample testing or cross-validation approaches.

## 2 Materials and methods

### 2.1 Study population

This study was designed as a retrospective cohort study, including patients diagnosed with RA who were admitted to and followed up in the Department of Rheumatology and Immunology of our hospital between September 1, 2022, and June 30, 2024. Clinical data and laboratory parameters of the enrolled patients were retrospectively extracted from the hospital’s electronic medical record (EMR) system and imaging/laboratory reporting databases. All data were carefully verified by the investigators and subsequently de-identified to ensure patient confidentiality before being used for statistical analysis.

### 2.2 Inclusion criteria

Patients were eligible for inclusion if they met all of the following criteria: age ≥ 18 years; a confirmed diagnosis of RA made by a rheumatologist during the study period according to the 2010 ACR/EULAR classification criteria for RA; had undergone at least one dual-energy X-ray absorptiometry (DXA) scan during or prior to enrollment with available and traceable BMD results for the lumbar spine (L1–L4) or hip (femoral neck/total hip); and had complete electronic medical records containing the key clinical and laboratory variables required for the study, including documented blood sampling time and fasting status.

### 2.3 Exclusion criteria

To minimize confounding and interference, the following patients were excluded: those with comorbid systemic diseases that could significantly affect bone metabolism (e.g., primary hypo-/hyperparathyroidism, severe chronic kidney disease with eGFR < 30 mL/min/1.73 m^2^ or CKD stage IV–V, active thyroid disorders, Cushing’s syndrome, uncontrolled diabetic ketoacidosis, or malignancies); patients who had received potent anti-resorptive or anti-osteoporotic therapy (e.g., denosumab, intravenous bisphosphonates) within the past 12 months; pregnant or lactating women; patients with pre-existing severe skeletal deformities or conditions that could compromise DXA accuracy; and those with incomplete clinical or follow-up data, making it impossible to determine osteoporosis outcomes or key covariates.

### 2.4 Outcome definition

Osteoporosis was defined according to DXA results, following the diagnostic criteria established by the World Health Organization. Specifically, a T-score ≤ −2.5 at either the lumbar spine (L1–L4) or femoral neck—whichever represented the lowest and most clinically relevant value—was classified as osteoporosis, whereas a T-score between −1.0 and −2.5 was defined as osteopenia. In addition, patients presenting with fragility fractures consistent with clinical and densitometric findings were also included in the osteoporosis group.

### 2.5 Data collection and measurement methods

All data in this study were retrospectively extracted from the hospital’s EMR system, laboratory information system, and imaging archives. Demographic and general clinical variables included sex, age at enrollment, disease duration (in months), BMI, smoking history, and histories of hypertension and diabetes. Autoimmune serological markers such as AKA, ANA, SSA (60 and 52 kD), and SSB were recorded as positive or negative. All demographic and medical history data were obtained from outpatient or inpatient records. Age was calculated as the age on the date of the DXA scan or baseline evaluation, disease onset age was defined as the time of first RA-related symptoms or confirmed diagnosis, and disease duration was recorded in months.

Assessment of disease activity and functional status included 28-tender joint count (TJC), 28-swollen joint count (SJC), patient’s pain visual analog scale (VAS), patient global assessment (PaGA), physician global assessment (MDGA), Disease Activity Score-28 (DAS28), Clinical Disease Activity Index (CDAI), Simplified Disease Activity Index (SDAI), and HAQ-DI. TJC and SJC were assessed and documented by rheumatologists following standard 28-joint evaluation protocols; VAS and MDGA were measured using a 0–100 mm scale. The formulas used were as follows: DAS28-ESR = 0.56 × √(TJC28) + 0.28 × √(SJC28) + 0.70 × ln(ESR) + 0.014 × VAS; CDAI = TJC + SJC + PaGA/10 + MDGA/10; pain VAS (0–10 cm) = PaGA/10; SDAI = TJC + SJC + PaGA/10 + MDGA/10 + CRP. HAQ-DI scores were extracted from clinical records. All dynamic and laboratory parameters were selected based on the results obtained closest in time to the baseline DXA examination.

Medication exposure and biochemical measurements were conducted following standardized protocols. Medication exposure included non-steroidal anti-inflammatory drugs (NSAIDs), glucocorticoids (daily and cumulative doses were recorded as prednisone-equivalent doses, with long-term use defined as ≥ 5 mg/day for ≥ 3 months based on predefined thresholds), conventional synthetic disease-modifying antirheumatic drugs (csDMARDs, such as methotrexate and azathioprine), and biologic agents (bDMARDs, such as TNF inhibitors and IL-6 inhibitors). All medication data were extracted from prescription records or outpatient/inpatient documentation.

Laboratory and biochemical parameters included hematological indices (WBC, monocyte count, RBC, Hb, mean platelet volume, etc.), inflammatory markers (ESR, CRP), rheumatologic biomarkers (RF), hepatic, renal, and metabolic indicators (AST, ALT, albumin, BUN, creatinine, uric acid), muscle enzyme and cellular metabolism indicators (LDH, CK), electrolytes and minerals (Ca, P), bone metabolism–related markers (ALP), lipid profile and apolipoproteins [TC, TG, HDL-C, LDL-C, ApoA1, ApoB, and lipoprotein(a)], and FFA.

The ApoA1/ApoB ratio was calculated directly from the measured concentrations. Derived hematologic ratios included the neutrophil-to-lymphocyte ratio (NLR), platelet-to-lymphocyte ratio (PLR), and monocyte-to-lymphocyte ratio (MLR), computed as neutrophil/lymphocyte, platelet/lymphocyte, and monocyte/lymphocyte ratios, respectively. All derived indices were calculated using absolute cell counts obtained from laboratory tests performed closest in time to the baseline DXA measurement.

All data were independently entered and cross-checked by two researchers, with outliers and missing values verified on a case-by-case basis. Missing data were handled according to predefined thresholds: variables with < 5% missing values were analyzed using a complete-case approach; those with 5–20% missing values were considered for multiple imputation; variables with > 20% missing data were excluded from model construction. A complete summary of missing data proportions for all candidate variables is provided in Supplementary Table S1.

### 2.6 Statistical analysis

The Shapiro-Wilk test was used to assess the normality of continuous variables. Normally distributed data are presented as mean ± standard deviation (SD), while non-normally distributed data are expressed as median and interquartile range (IQR). For skewed distributions, group comparisons were performed using the Wilcoxon-Mann-Whitney U test. Categorical variables are summarized as frequencies (percentages) and compared using Pearson’s chi-square test or Fisher’s exact test, as appropriate. Spearman correlation analysis and Belsley collinearity diagnostics were conducted to evaluate multicollinearity among covariates. A two-sided *p*-value < 0.05 was considered statistically significant.

To develop the predictive nomogram, univariable and multivariable logistic regression analyses were performed using a stepwise backward selection approach, and odds ratios (ORs) with 95% confidence intervals (CIs) were calculated. Variables with *p* < 0.05 in univariable analysis were considered candidates for entry into the multivariable model. The significance threshold for variable retention in the final model was set at *p* < 0.05. This analysis aimed to identify a simplified model in the development cohort. The nomogram was then constructed based on the identified predictive factors. Model performance was evaluated in the training cohort and subsequently validated in a temporal validation cohort comprising patients enrolled later in the study period (internal temporal validation). Discrimination was evaluated by calculating the area under the receiver operating characteristic curve (AUROC). Calibration curves were generated to assess agreement between predicted and observed outcomes, with goodness-of-fit evaluated using the Hosmer-Lemeshow test. Clinical utility and net benefit were estimated via decision curve analysis (DCA) and clinical impact curve analysis (CICA). Based on total nomogram scores, patients were stratified into low-, medium-, and high-risk subgroups for osteoporosis, forming a new risk stratification system. All statistical analyses were performed using SPSS version 26.0 and Stata version 16.0.

## 3 Results

### 3.1 Study population

**FIGURE 1:**
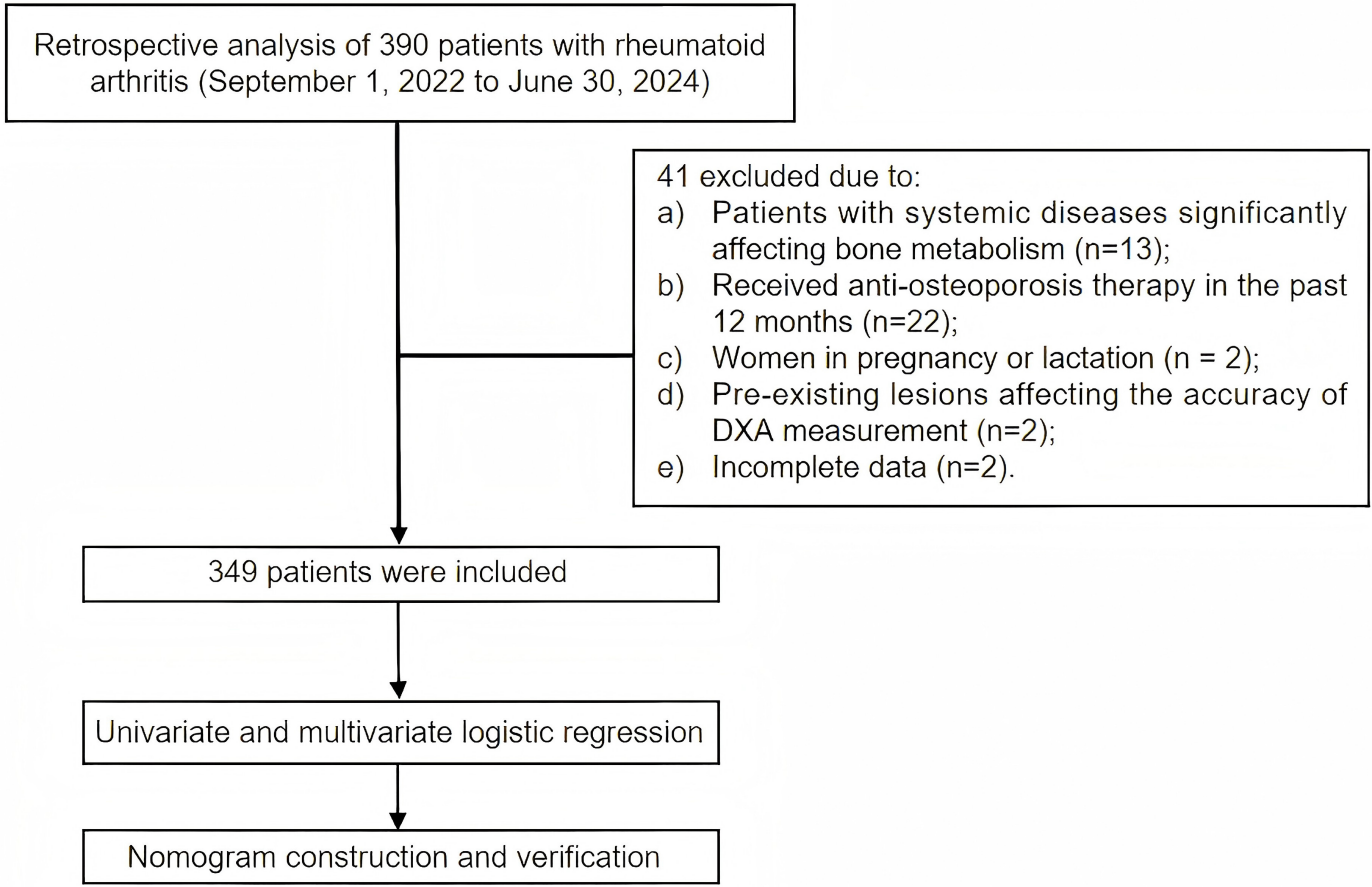
Flowchart of patient recruitment and study population selection.

Of the 390 patients initially screened, 41 were excluded based on predefined criteria, leaving a total of 349 patients for analysis, of whom 132 (37.82%) were diagnosed with osteoporosis. The study flowchart is presented in Figure 1.

Table 1 summarizes the characteristics of the study population and compares patients with and without osteoporosis. Among the 349 RA patients, the prevalence of osteoporosis was 88.64% in females (*n* = 117) and 11.36% in males (*n* = 15). Compared with patients without osteoporosis, those with osteoporosis were more likely to be female, older, and have a longer disease duration. Moreover, patients with osteoporosis exhibited higher values for TJC, SJC, VAS for pain, PaGA, MDGA, DAS28, CDAI, SDAI, HAQ-DI, ALP, ApoA1/ApoB ratio, and FFA, whereas RBC, Hb, Alb, Cr, and BMI were lower compared to non-osteoporotic patients.

**TABLE 1:** Baseline characteristics of RA patients with and without osteoporosis.

Variables	Overall	Non-osteoporosis	Osteoporosis	*p*
	*n* = 349	*n* = 217	*n* = 132	
Gender, n (%)				
Male	99 (28.37)	84 (38.71)	15 (11.36)	< 0.001
Female	250 (71.63)	133 (61.29)	117 (88.64)	
Whether to use NSAIDs, n (%)				
No	34 (9.74)	20 (9.22)	14 (10.61)	
Yes	315 (90.26)	197 (90.78)	118 (89.39)	0.812
Whether to use hormone drugs, n (%)				
No	215 (61.60)	141 (64.98)	74 (56.06)	
Yes	134 (38.40)	76 (35.02)	58 (43.94)	0.122
Whether to use immune inhibitors, n (%)				
No	155 (44.41)	95 (43.78)	60 (45.45)	
Yes	194 (55.59)	122 (56.22)	72 (54.55)	0.846
Whether to use biological agents, n (%)				
No	313 (89.68)	195 (89.86)	118 (89.39)	
Yes	36 (10.32)	22 (10.14)	14 (10.61)	1
AKA, n (%)				
Negative	127 (36.39)	77 (35.48)	50 (37.88)	
Positive	222 (63.61)	140 (64.52)	82 (62.12)	0.737
ANA, n (%)				
Negative	281 (80.52)	176 (81.11)	105 (79.55)	
Positive	68 (19.48)	41 (18.89)	27 (20.45)	0.828
SSA60KD, n (%)				
Negative	325 (93.12)	205 (94.47)	120 (90.91)	
Positive	24 (6.88)	12 (5.53)	12 (9.09)	0.291
SSA52KD, n (%)				
Negative	317 (90.83)	201 (92.63)	116 (87.88)	
Positive	32 (9.17)	16 (7.37)	16 (12.12)	0.194
SSB, n (%)				
Negative	338 (96.85)	209 (96.31)	129 (97.73)	
Positive	11 (3.15)	8 (3.69)	3 (2.27)	0.677
ILD, n (%)				
Negative	316 (90.54)	197 (90.78)	119 (90.15)	
Positive	33 (9.46)	20 (9.22)	13 (9.85)	0.994
Hypertension, n (%)				
No	241 (69.05)	149 (68.66)	92 (69.70)	
Yes	108 (30.95)	68 (31.34)	40 (30.30)	0.934
Diabetes, n (%)				
No	308 (88.25)	194 (89.40)	114 (86.36)	
Yes	41 (11.75)	23 (10.60)	18 (13.64)	0.495
History of smoking, n (%)				
No	262 (75.07)	162 (74.65)	100 (75.76)	
Yes	87 (24.93)	55 (25.35)	32 (24.24)	0.918
History of smoking, n (%)				
Age (median [IQR])	62.000 [56.000, 69.000]	59.000 [54.000, 67.000]	66.500 [58.750, 72.000]	< 0.001
Course of disease (months) (median [IQR])	48.000 [6.000, 120.000]	36.000 [5.000, 120.000]	60.000 [12.000, 121.000]	0.007
TJC (median [IQR])	7.000 [4.000, 12.000]	6.000 [4.000, 10.000]	8.500 [4.000, 14.000]	0.001
SJC (median [IQR])	3.000 [1.000, 5.000]	2.000 [1.000, 4.000]	4.000 [2.000, 6.000]	0.001
VAS (median [IQR])	5.000 [3.000, 6.000]	4.000 [3.000, 5.000]	6.000 [4.000, 7.000]	< 0.001
PaGA (median [IQR])	50.000 [30.000, 60.000]	40.000 [30.000, 50.000]	60.000 [40.000, 70.000]	< 0.001
MDGA (median [IQR])	40.000 [30.000, 60.000]	40.000 [30.000, 50.000]	50.000 [40.000, 60.000]	< 0.001
DAS28 (median [IQR])	4.650 [4.019, 5.328]	4.471 [3.947, 5.139]	4.977 [4.284, 5.560]	< 0.001
CDAI (median [IQR])	19.000 [13.000, 27.000]	17.000 [12.000, 23.000]	23.000 [16.000, 31.000]	< 0.001
SDAI (median [IQR])	38.500 [24.600, 71.200]	35.600 [22.270, 70.300]	43.050 [29.830, 73.725]	0.045
HAQ-DI (median [IQR])	0.750 [0.450, 1.200]	0.600 [0.350, 0.950]	1.050 [0.650, 1.450]	< 0.001
ESR (median [IQR])	50.000 [28.000, 74.000]	48.000 [25.000, 70.000]	51.500 [34.000, 78.000]	0.077
CRP (median [IQR])	15.400 [5.530, 50.300]	15.300 [5.400, 47.300]	15.400 [5.722, 52.825]	0.811
RF (median [IQR])	143.000 [34.600, 492.000]	142.000 [32.800, 522.000]	160.500 [37.500, 454.250]	0.860
WBC (median [IQR])	6.700 [5.100, 8.300]	6.700 [5.300, 8.500]	6.500 [4.975, 8.025]	0.147
Monocyte Count, (median [IQR])	0.460 [0.350, 0.610]	0.480 [0.370, 0.620]	0.420 [0.320, 0.582]	0.056
RBC (median [IQR])	3.920 [3.560, 4.210]	3.960 [3.570, 4.320]	3.845 [3.520, 4.130]	0.018
Hb (median [IQR])	114.000 [102.000, 125.000]	116.000 [104.000, 125.000]	112.000 [98.000, 121.250]	0.005
MPV (median [IQR])	10.300 [9.500, 11.200]	10.200 [9.500, 11.100]	10.500 [9.600, 11.300]	0.057
NLR (median [IQR])	2.941 [2.040, 4.390]	2.901 [2.110, 4.345]	2.953 [1.930, 4.494]	0.859
PLR (median [IQR])	173.762 [126.291, 250.000]	171.812 [126.471, 239.831]	180.811 [125.681, 263.733]	0.449
MLR (median [IQR])	0.321 [0.226, 0.420]	0.329 [0.241, 0.420]	0.316 [0.204, 0.419]	0.273
AST (median [IQR])	19.000 [15.000, 24.000]	18.000 [15.000, 24.000]	19.000 [16.000, 25.000]	0.179
ALT (median [IQR])	14.000 [10.000, 21.000]	15.000 [10.000, 22.000]	14.000 [9.000, 19.250]	0.197
Alb (median [IQR])	34.600 [31.800, 37.500]	35.100 [31.900, 37.900]	33.950 [31.350, 36.525]	0.021
Alkaline phosphatase (median [IQR])	90.000 [73.000, 107.000]	86.000 [72.000, 103.000]	92.500 [76.000, 116.000]	0.037
LDH (median [IQR])	209.000 [178.000, 248.000]	207.000 [179.000, 240.000]	209.000 [177.500, 254.000]	0.343
CK (median [IQR])	37.000 [25.000, 54.000]	38.000 [25.000, 54.000]	33.000 [24.000, 54.250]	0.490
BUN (median [IQR])	5.700 [4.500, 7.100]	5.800 [4.600, 7.200]	5.650 [4.500, 7.000]	0.403
Cr (median [IQR])	53.000 [46.000, 63.000]	54.000 [47.000, 65.000]	51.000 [43.000, 59.250]	0.011
UA (median [IQR])	284.000 [234.000, 347.000]	284.000 [236.000, 352.000]	284.500 [232.000, 339.750]	0.708
Ca (median [IQR])	2.200 [2.140, 2.270]	2.210 [2.150, 2.270]	2.190 [2.130, 2.270]	0.116
P (median [IQR])	1.210 [1.080, 1.330]	1.210 [1.090, 1.320]	1.210 [1.070, 1.350]	0.905
TC (median [IQR])	4.300 [3.700, 5.100]	4.200 [3.700, 5.100]	4.300 [3.800, 5.025]	0.394
TG (median [IQR])	1.090 [0.820, 1.490]	1.110 [0.820, 1.480]	1.075 [0.860, 1.510]	0.711
HDLC (median [IQR])	1.120 [0.930, 1.310]	1.110 [0.910, 1.280]	1.140 [0.950, 1.332]	0.123
LDLC (median [IQR])	2.860 [2.370, 3.370]	2.880 [2.410, 3.370]	2.805 [2.357, 3.350]	0.410
ApoA1 (median [IQR])	1.270 [1.090, 1.430]	1.270 [1.080, 1.430]	1.275 [1.120, 1.460]	0.240
ApoB (median [IQR])	0.760 [0.620, 0.920]	0.760 [0.650, 0.930]	0.750 [0.610, 0.880]	0.254
ApoA1/ApoB (median [IQR])	1.700 [1.300, 2.100]	1.600 [1.300, 2.000]	1.900 [1.475, 2.300]	< 0.001
Lpa (median [IQR])	203.500 [98.700, 400.000]	197.300 [96.000, 394.700]	219.850 [113.150, 401.675]	0.409
FFA (median [IQR])	0.350 [0.220, 0.480]	0.320 [0.220, 0.440]	0.390 [0.248, 0.522]	0.005
BMI (median [IQR])	22.600 [20.703, 25.110]	22.893 [20.937, 25.403]	22.240 [20.269, 24.582]	0.023

### 3.2 Model development and validation

A total of 250 patients were included in the training cohort, of whom 92 (36.8%) had osteoporosis, while 99 patients were included in the validation cohort, with 40 (40.4%) cases of osteoporosis. The clinical and demographic characteristics of the training and validation cohorts are summarized in Supplementary Table S2, showing no statistically significant differences between the two groups.

Logistic regression analyses were performed to identify risk factors associated with osteoporosis. In univariate logistic regression, potential risk factors included female sex, older age, longer disease duration, higher TJC, SJC, VAS for pain, PaGA, MDGA, DAS28, CDAI, HAQ-DI, ALP, ApoA1/ApoB ratio, and FFA, whereas RBC and BMI were lower in patients with osteoporosis (*p* < 0.05) (Table 2). Multivariate logistic regression further identified female sex, HAQ-DI, ALP, ApoA1/ApoB ratio, FFA, and decreased BMI as independent predictors of osteoporosis in RA patients.

**TABLE 2:** Univariate and multivariate logistic regression analyses of risk factors for osteoporosis in patients with RA.

Characteristics	Univariate analysis			Multivariate analysis		
	OR	95%CI	*P*	OR	95%CI	*P*
Gender						
Male	Ref					
Female	0.20	0.11–0.37	< 0.001	0.12	0.06–0.27	< 0.001
Age	1.06	1.04–1.09	< 0.001	1.06	0.85–1.33	0.595
Course of disease (months)	1.02	1.01–1.06	0.040	1.00	0.99–1.02	0.642
TJC	1.06	1.02–1.09	< 0.001	0.95	0.87–1.03	0.196
SJC	1.07	1.01–1.13	0.020	1.00	0.90–1.10	0.919
VAS	1.45	1.27–1.66	< 0.001	1.29	0.97–1.73	0.079
PaGA	1.04	1.02–1.05	< 0.001	NA	NA	NA
MDGA	1.04	1.03–1.06	< 0.001	0.99	0.96–1.03	0.712
DAS28	1.45	1.16–1.81	< 0.001	1.36	0.75–2.46	0.313
CDAI	1.04	1.02–1.06	< 0.001	NA	NA	NA
SDAI	1.01	0.98–1.01	0.190	NA	NA	NA
HAQ	3.80	2.42–5.94	< 0.001	2.35	1.09–5.10	0.030
RBC	0.58	0.39–0.87	0.010	1.20	0.65–2.19	0.562
Hb	0.99	0.96–1.04	0.310	NA	NA	NA
Alb	0.96	0.92–1.01	0.090	NA	NA	NA
Alkaline phosphatase	1.02	1.01–1.04	0.030	1.02	1.01–1.03	0.005
Cr	0.99	0.98–1.01	0.660	NA	NA	NA
ApoA1/ApoB	1.86	1.28–2.72	< 0.001	1.68	1.02–2.78	0.043
FFA	6.43	1.94–21.31	< 0.001	5.26	1.14–24.23	0.033
BMI	0.91	0.85–0.98	0.010	0.91	0.83–0.99	0.045

**FIGURE 2:**
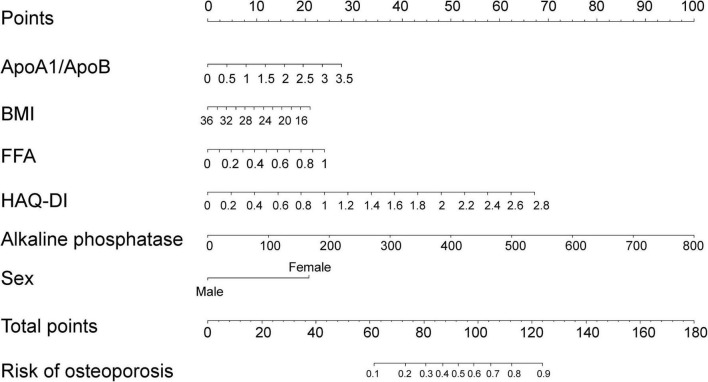
Nomogram for predicting the risk of osteoporosis in patients with RA.

These six variables were incorporated into the model development. Based on these independent predictors, a nomogram was constructed to estimate the probability of osteoporosis in RA patients (Figure 2). Each predictor is assigned a score according to the points indicated in the first row of the nomogram. The total score is calculated by summing the points for all predictors. By locating the total score on the total points axis, the corresponding probability of developing osteoporosis can be estimated.

### 3.3 Validation of the nomogram model

**FIGURE 3:**
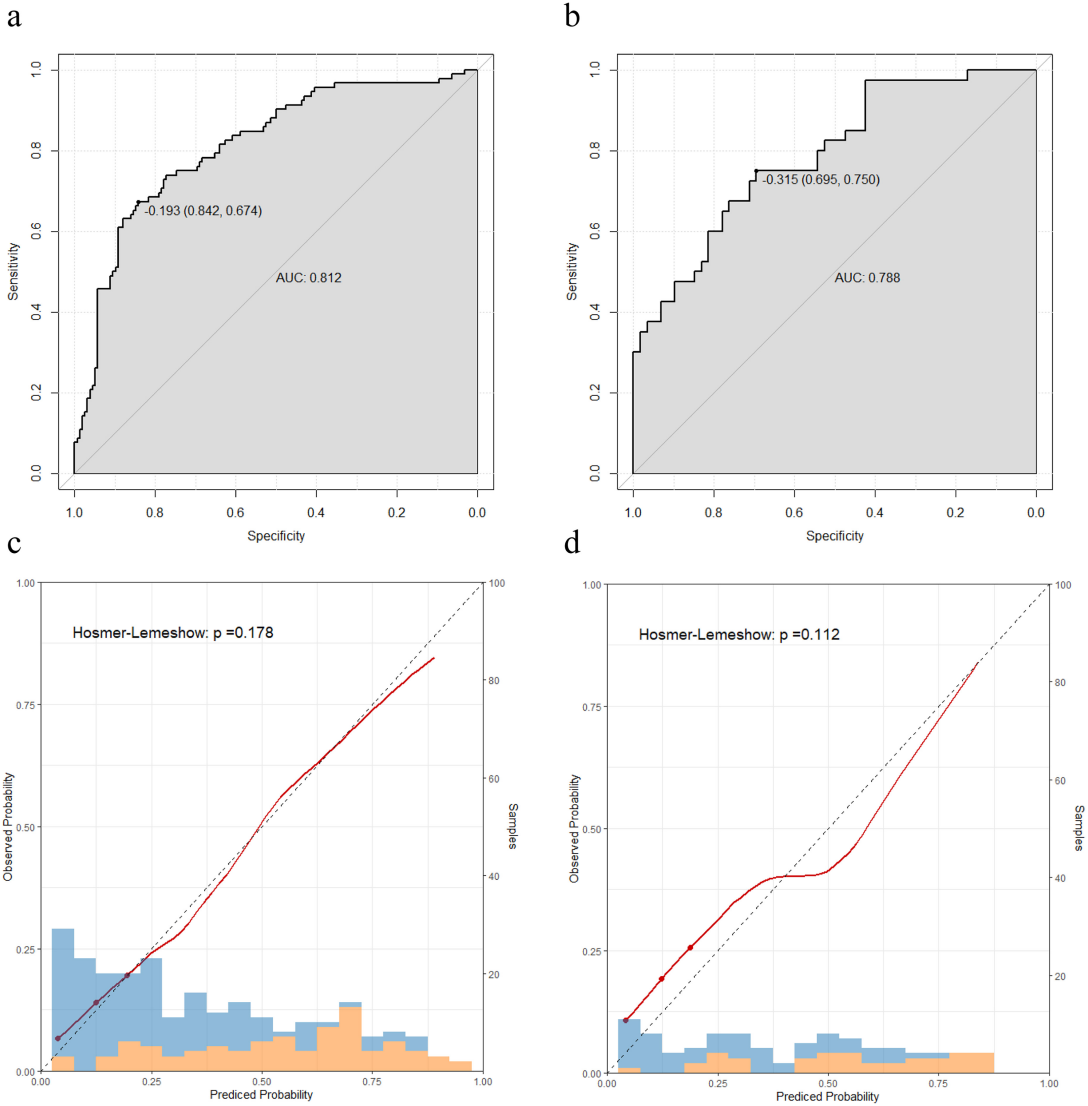
Validation of the nomogram for predicting osteoporosis in RA patients. **(a)** ROC curve for the training cohort. **(b)** ROC curve for the validation cohort. **(c)** Calibration curve for the training cohort. **(d)** Calibration curve for the validation cohort.

The predictive performance of the nomogram for osteoporosis was evaluated in both the training and validation cohorts using ROC curve analysis. The AUC was 0.812 in the training cohort (Figure 3A) and 0.788 in the validation cohort (Figure 3B), indicating good discriminative ability of the model. To further assess calibration, bootstrap-resampled calibration curves were generated for the training cohort (Figure 3C) and the validation cohort (Figure 3D). The x-axis represents the predicted risk of osteoporosis, while the y-axis represents the observed incidence. The diagonal dashed line corresponds to a perfect prediction model. The closer the calibration curve aligns with the diagonal line, the higher the predictive accuracy of the nomogram. Both curves demonstrated slight linear deviations, indicating excellent calibration performance. Moreover, Hosmer-Lemeshow tests confirmed no statistically significant differences between predicted and observed risks (all *P* > 0.05).

### 3.4 Clinical application

**FIGURE 4:**
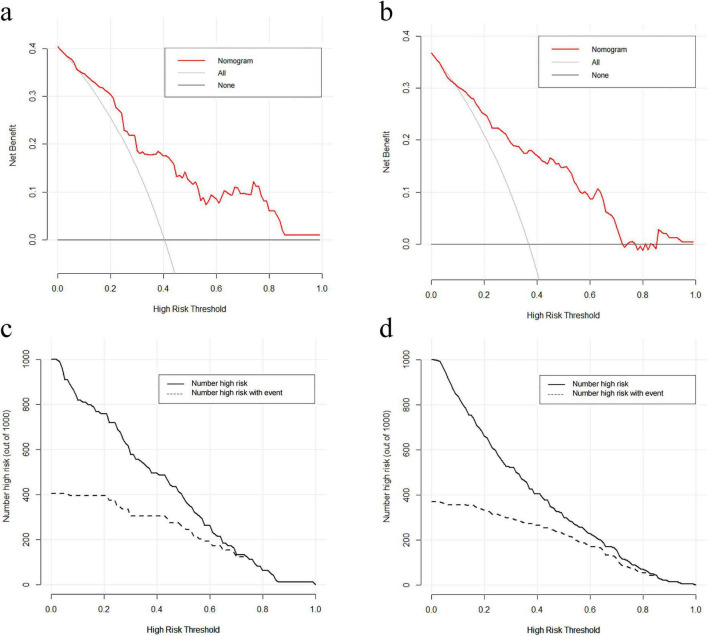
DCA and CICA of the osteoporosis risk nomogram in RA patients. **(a,b)** DCA in the training cohort and validation cohort, showing net benefit across a range of threshold probabilities. **(c,d)** CICA in the training cohort and validation cohort, illustrating the number of patients classified as high risk versus the number of true high-risk patients at each threshold.

The DCA of the osteoporosis risk nomogram is shown in Figure 4. The y-axis represents the net benefit, and the x-axis represents the threshold probability for developing osteoporosis. The diagonal line indicates a scenario in which all patients receive intervention, whereas the horizontal line represents a scenario in which no patients receive intervention, resulting in a net benefit of zero. Net benefit is calculated by subtracting the proportion of false-positive patients from the proportion of true-positive patients, weighted according to the relative harm of missing treatment versus the negative consequences of unnecessary treatment. The threshold probability reflects the likelihood of osteoporosis occurrence and helps guide clinicians in deciding whether to initiate intervention. In both the training and validation cohorts, most of the curves for predicted threshold probabilities lie above the two extreme lines across the 0–1 probability range, indicating substantial clinical utility of the model (Figures 4A,B).

The CICA, an extension of DCA, visually demonstrates the clinical applicability of the predictive model at different risk thresholds. The x-axis represents various risk thresholds used to classify patients as high-risk. The solid line indicates the number of patients predicted to be at high risk at each threshold, while the dashed line represents the actual number of true high-risk patients (those who truly developed osteoporosis). The close alignment of the two curves indicates that the model accurately identifies high-risk patients, supporting its high clinical utility (Figures 4C,D).

### 3.5 Risk stratification analysis

**FIGURE 5:**
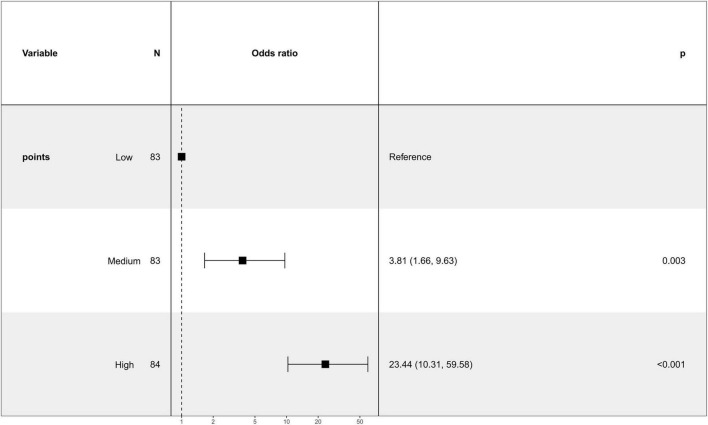
Risk stratification based on nomogram scores.

Patients were stratified into low-, medium-, and high-risk groups according to their total nomogram scores. Based on the distribution of total points in the training cohort, cutoff values were determined using the 33.3rd and 66.7th percentiles, corresponding to total points of < 54.20 for the low-risk group, 54.20–77.70 for the medium-risk group, and > 77.70 for the high-risk group. Using the low-risk group as the reference, both the medium- and high-risk groups demonstrated odds ratios greater than 1 for the occurrence of osteoporosis (medium-risk: OR = 3.81, 95% CI: 1.66–9.63, *p* = 0.003; high-risk: OR = 23.44, 95% CI: 10.31–59.58, *p* < 0.001), indicating that these differences were statistically significant (Figure 5). These findings confirm that the nomogram-derived score effectively discriminates between risk categories and can meaningfully stratify RA patients by their probability of developing osteoporosis, supporting its potential utility for guiding clinical decision-making and prioritizing preventative interventions.

Patients were stratified into low-, medium-, and high-risk groups according to their total nomogram scores. Using the low-risk group as the reference, both the medium- and high-risk groups demonstrated odds ratios greater than 1 for the occurrence of osteoporosis, with *p* < 0.05, indicating that these differences were statistically significant (Figure 5). These findings confirm that the nomogram-derived score effectively discriminates between risk categories and can meaningfully stratify RA patients by their probability of developing osteoporosis, supporting its potential utility for guiding clinical decision-making and prioritizing preventative interventions.

## 4 Discussion

In this single-center retrospective cohort study, we developed and externally validated a nomogram to predict the risk of osteoporosis in patients with RA, and identified several independent predictors. Female sex, higher HAQ-DI scores, elevated ALP, an increased ApoA1/ApoB ratio, higher FFA levels, and lower BMI were all independently associated with osteoporosis. The nomogram demonstrated good discrimination and calibration, indicating robust predictive performance, and its inputs are readily available in routine clinical practice. Our nomogram offers complementary advantages by incorporating RA-specific continuous variables and metabolic biomarkers, thereby capturing pathophysiological heterogeneity and enabling more individualized risk stratification. It is not intended to replace FRAX, but rather to serve as an adjunct for RA-specific osteoporosis screening, particularly when detailed RA-related factors are relevant.

Female sex, particularly postmenopausal status, is a well-established risk factor for osteoporosis in RA patients, with postmenopausal women exhibiting markedly higher osteoporosis rates than men and premenopausal women (17). This increased risk is primarily mediated by estrogen deficiency, which accelerates bone resorption and interacts with inflammatory pathways to amplify bone loss (18, 19). Estrogen deprivation enhances osteoclast activity and upregulates pro-resorptive cytokines (TNF-α, IL-1, IL-6, RANKL), while also attenuating the bone’s adaptive response to mechanical loading (20, 21). Consequently, osteoporosis is particularly prevalent in postmenopausal RA patients, with some studies reporting rates approaching 50% (22). These observations underscore the importance of incorporating menopausal status into risk stratification and preventive strategies for RA-associated osteoporosis.

The HAQ-DI reflects the daily functional capacity and activity level of RA patients, with functional limitations contributing to bone loss through multiple mechanisms (23). First, reduced physical activity and mechanical loading directly diminish bone stimulation, suppressing the mechanosensitive Wnt/β-catenin signaling pathway in osteocytes. This pathway primarily promotes bone anabolic metabolism, with osteocytes serving as its key coordinators. Activation of Wnt/β-catenin in osteocytes modulates a complex network involving Notch signaling and the endogenous inhibitor sclerostin, asymmetrically enhancing both bone formation and resorption, ultimately resulting in net bone gain (24). Long-term inhibition of this pathway leads to decreased bone formation and progressive bone loss (25). Second, chronic functional limitation is often accompanied by declines in muscle mass and strength, contributing to sarcopenia and disrupting muscle-bone crosstalk, which further compromises bone strength (26). Thus, HAQ-DI serves as a composite indicator that not only reflects behavioral and mechanical loading-related risk but also captures the indirect effects of chronic inflammation and treatment burden on bone metabolism.

ALP was identified as an independent predictor of osteoporosis in our RA cohort. ALP, particularly its bone-specific isoform produced by osteoblasts, serves as a marker of bone turnover and mineralization (27). In high bone turnover states, ALP levels typically increase, reflecting enhanced osteoblast activity; however, when bone resorption exceeds formation, net bone loss may still occur (28). Several population-based studies have reported associations between elevated ALP and reduced BMD, supporting its biological plausibility as a predictor (29). In RA patients with osteoporosis, significantly elevated bone-specific ALP levels have been documented (30). Clinically, ALP is routinely measured, inexpensive, and readily available, making it a practical addition to prediction models. However, ALP elevations may be influenced by confounders such as liver function or medication use, and future research should explore whether incorporating additional bone turnover markers (e.g., P1NP, β-CTX) could improve predictive performance (31).

In the present study, an elevated ApoA1/ApoB ratio was positively associated with osteoporosis risk. Multiple large-scale investigations have reported findings consistent with our observations. Yamaguchi et al. first hypothesized that lipid metabolism disturbances may participate in the pathogenesis of osteoporosis and demonstrated a positive association between high-density lipoprotein cholesterol (HDL-C) levels and osteoporosis risk in 214 postmenopausal Asian women (32). Subsequently, Jeong et al. confirmed in 10,402 Korean women that higher HDL-C levels were associated with increased lumbar osteoporosis risk in postmenopausal women (33). Similarly, Li et al. demonstrated in 790 Chinese postmenopausal women that elevated HDL-C was significantly associated with a higher probability of osteoporosis (34). Most notably, Chen et al. provided genetic evidence through a genome-wide association study, confirming a positive causal relationship between HDL-C and osteoporotic fracture risk in older adults (35). A large cross-sectional study utilizing NHANES data (*N* = 7,743) also demonstrated that, after comprehensive multivariable adjustment, higher ApoA1 levels were significantly associated with increased osteoporosis risk (OR = 2.289, 95% CI: 1.350–3.881, *p* = 0.002) (36). Additionally, a study investigating ApoB-100 in postmenopausal women revealed a significant independent negative correlation with lumbar bone mineral density (β = −6.37, 95% CI: −9.26 to −3.49), with mediation analysis indicating that inflammatory pathways partially mediated this association (37). This finding underscores the mechanistic importance of inflammation in the relationship between apolipoproteins and bone metabolism.

Elevated free fatty acids (FFA) were identified as an independent predictor of osteoporosis in our RA cohort. FFAs may promote bone loss through multiple mechanisms: inducing lipotoxicity in osteoblasts, activating inflammatory pathways (NF-κB, MAPK) that enhance osteoclast differentiation, and stimulating pro-inflammatory cytokines such as TNF-α and IL-6 (16, 38). A recent retrospective study of 276 patients demonstrated that individuals with osteoporosis had significantly higher FFA levels than those with normal bone mass, and combining FFA with apolipoprotein markers improved predictive accuracy over single markers alone (39). These findings suggest that FFAs may contribute to osteoporosis pathogenesis partly through inflammation-mediated pathways, and joint assessment of lipid metabolism and inflammatory markers could enhance early risk prediction (40). As routinely measured clinical parameters, FFAs represent a practical addition to osteoporosis risk assessment in RA patients.

Low BMI has been consistently associated with osteoporosis in both general populations and RA cohorts, with mechanisms primarily involving insufficient mechanical loading, nutritional and endocrine factors, and muscle–bone interactions. A study of 304 RA patients undergoing DXA assessment found that despite advances in RA management, the prevalence of osteoporosis remained significantly higher in RA patients (29.9%) compared to the general population (17.4%), with BMI identified as an independent predictor (35). Reduced BMI reflects diminished adipose and lean body mass, leading to decreased mechanical loading on the skeleton and suppression of osteogenic signaling (41). Concurrently, lower adipose tissue decreases secretion of leptin and estrogen, further impairing bone metabolic homeostasis (42). Moreover, insufficient nutritional reserves and enhanced bone resorption under chronic inflammatory conditions contribute to increased osteoporosis susceptibility in RA patients. In summary, in RA, low BMI not only signals reduced mechanical loading, inadequate nutritional and endocrine support, and muscle–bone imbalance but also often reflects higher disease activity and metabolic consumption. Thus, BMI is a biologically and clinically plausible independent predictor of osteoporosis. In clinical practice, BMI should be integrated with body composition, inflammation control, and medication history to guide assessment and intervention strategies aimed at minimizing the risk of osteoporosis and fracture.

Our study, based on a single-center retrospective cohort, is the first in China to systematically construct and validate a risk prediction nomogram for osteoporosis in RA patients, integrating multidimensional indicators including sex, HAQ-DI, ALP, ApoA1/ApoB ratio, FFA, and BMI. Compared with previous models that relied on single risk factors such as age or BMI, this nomogram more accurately reflects the complex pathophysiological state of RA patients, enabling individualized assessment of osteoporosis risk. Internal validation demonstrated excellent discrimination and calibration, indicating high predictive accuracy and clinical utility. The model can serve as an effective tool for clinicians to identify high-risk patients early and to guide personalized prevention and treatment strategies. Consistent with prior studies, factors such as female sex, low BMI, and dysregulated inflammation and metabolism are fundamental mechanisms underlying osteoporosis in RA, further supporting the biological plausibility of the model’s predictions.

The model demonstrated moderate discrimination (AUC 0.812 in the training cohort and 0.788 in the validation cohort) and acceptable calibration in internal validation. Nevertheless, several limitations should be acknowledged. First, as a single-center retrospective study with a limited sample size, it may be subject to selection and information biases. Second, while we performed internal temporal validation by splitting the cohort based on enrollment time, this does not constitute true external validation. The validation cohort was derived from the same institution and patient population, and therefore the model’s performance in entirely new cohorts from different centers or geographic regions remains unknown. Consequently, external validation in multicenter prospective cohorts is warranted to confirm the generalizability and transportability of the model across diverse clinical settings and populations. Third, BMD assessment relied solely on DXA measurements without incorporating bone turnover markers (e.g., procollagen type I N-terminal propeptide, β-C-terminal telopeptide, or tartrate-resistant acid phosphatase 5b) or imaging-based bone microstructure parameters (e.g., trabecular bone score, high-resolution peripheral quantitative computed tomography, or quantitative ultrasound parameters). The absence of these measures represents a significant limitation, as they could provide complementary information about bone quality, microarchitecture, and dynamic remodeling activity that DXA-derived BMD alone cannot capture. Fourth, several clinically important osteoporosis risk factors in RA were not incorporated due to retrospective data limitations, including: (1) menopausal status; (2) prior or ongoing anti-osteoporotic therapy; (3) cumulative glucocorticoid exposure (assessed only as binary ever-use); (4) vitamin D status and calcium intake; (5) physical activity levels; and (6) family history of fracture. Omission of these variables may introduce bias and affect the estimated coefficients of included predictors. Finally, although the nomogram integrates multiple clinical and metabolic factors, it does not include inflammatory cytokines or molecular markers of bone remodeling pathways, leaving room for mechanistic refinement. Future research combining longitudinal follow-up and multi-omics data could enable the development of integrated AI-based predictive tools, bridging mechanistic insights and precision medicine applications. Overall, this study provides a novel quantitative tool for assessing osteoporosis risk in RA patients, offering a scientific basis for early intervention and personalized management.

## 5 Conclusion

Based on a single-center retrospective cohort, our study systematically developed and validated a nomogram for predicting osteoporosis risk in patients with RA, integrating multidimensional indicators including sex, HAQ-DI, ALP, ApoA1/ApoB ratio, FFA, and BMI. The results demonstrated that female sex, higher HAQ-DI scores, elevated ALP, increased ApoA1/ApoB ratio, elevated FFA, and lower BMI are independent predictors of osteoporosis in RA patients.

## Data Availability

The original contributions presented in the study are included in the article/Supplementary material, further inquiries can be directed to the corresponding author.

## References

[1] Smolen J Aletaha D McInnes I. Rheumatoid arthritis. *Lancet.* (2016) 388:2023–38. 10.1016/S0140-6736(16)30173-8 27156434

[2] Wysham K Baker J Narla R. Osteoporosis and fractures in rheumatoid arthritis - risk factors. *Best Pract Res Clin Rheumatol.* (2022) 36:101757. 10.1016/j.berh.2022.101757 36208961

[3] Wysham K Baker J Shoback D. Osteoporosis and fractures in rheumatoid arthritis. *Curr Opin Rheumatol.* (2021) 33:270–6. 10.1097/BOR.0000000000000789 33651725

[4] Moshayedi S Tasorian B Almasi-Hashiani A. The prevalence of osteoporosis in rheumatoid arthritis patient: a systematic review and meta-analysis. *Sci Rep.* (2022) 12:15844. 10.1038/s41598-022-20016-x 36151246 PMC9508181

[5] Zhang Z Ho S Chen Z Zhang C Chen Y. Reference values of bone mineral density and prevalence of osteoporosis in chinese adults. *Osteoporos Int.* (2014) 25:497–507. 10.1007/s00198-013-2418-2 23800746

[6] Haseltine K Chukir T Smith P Jacob J Bilezikian J Farooki A. Bone mineral density: clinical relevance and quantitative assessment. *J Nucl Med.* (2021) 62:446–54. 10.2967/jnumed.120.256180 33310738 PMC8049374

[7] Lems W Dijkmans B. Should we look for osteoporosis in patients with rheumatoid arthritis? *Ann Rheum Dis.* (1998) 57:325–7. 10.1136/ard.57.6.325 9771204 PMC1752623

[8] Nolla J Fiter J Gómez-Vaquero C Alegre J Valverde J Roig-Escofet D. Value of clinical factors in selecting postmenopausal women with rheumatoid arthritis for bone densitometry. *Ann Rheum Dis.* (2001) 60:799–801. 10.1136/ard.60.8.799 11454646 PMC1753792

[9] Kvien T Haugeberg G Uhlig T Falch J Halse J Lems W et al. Data driven attempt to create a clinical algorithm for identification of women with rheumatoid arthritis at high risk of osteoporosis. *Ann Rheum Dis.* (2000) 59:805–11. 10.1136/ard.59.10.805 11005782 PMC1753011

[10] Chen R Huang Q Chen L. Development and validation of machine learning models for prediction of fracture risk in patients with elderly-onset rheumatoid arthritis. *Int J Gen Med.* (2022) 15:7817–29. 10.2147/IJGM.S380197 36276661 PMC9581722

[11] Lee J Sung Y Choi C Cho S Bang S Choe J et al. The frequency of and risk factors for osteoporosis in Korean patients with rheumatoid arthritis. *BMC Musculoskelet Disord.* (2016) 17:98. 10.1186/s12891-016-0952-8 26912147 PMC4765070

[12] Yan X Xu Z Li S Yan L Lyu G Wang Z. Establishment and verification of an osteoporosis risk model in patients with rheumatoid arthritis: a valuable new model. *Arch Osteoporos.* (2021) 16:3. 10.1007/s11657-020-00867-5 33394305 PMC7782444

[13] Zeng T Tan L Yu J Wu Y. High density lipoprotein in rheumatoid arthritis: emerging role in predicting inflammation level and osteoporosis occurrence. *Scand J Clin Laboratory Invest.* (2020) 80:375–80. 10.1080/00365513.2020.1747109 32279574

[14] Jiang Z Yao X Lan W Ma W Yao X Fang T. Association of HDL and LDL levels with osteoporosis in rheumatoid arthritis: a retrospective cohort study. *Eur J Med Res.* (2024) 29:439. 10.1186/s40001-024-02013-0 39210479 PMC11360834

[15] Zhong L Li Q Jiang Y Cheng D Liu Z Wang B et al. The ApoB/ApoA1 ratio is associated with metabolic syndrome and its components in a chinese population. *Inflammation.* (2010) 33:353–8. 10.1007/s10753-010-9193-4 20213498

[16] Frommer K Hasseli R Schaffler A Lange U Rehart S Steinmeyer J et al. Free fatty acids in bone pathophysiology of rheumatic diseases. *Front Immunol.* (2019) 10:2757. 10.3389/fimmu.2019.02757 31849953 PMC6901602

[17] Raine C Giles I. What is the impact of sex hormones on the pathogenesis of rheumatoid arthritis? *Front Med.* (2022) 9:909879. 10.3389/fmed.2022.909879 35935802 PMC9354962

[18] Chen T Zhang Z Yang S Zhu Y. Frequency of osteoporosis in chinese patients with rheumatoid arthritis: a meta-analysis. *Arch Osteoporos.* (2023) 18:24. 10.1007/s11657-023-01212-2 36689130

[19] Islander U Jochems C Lagerquist M Forsblad-d’Elia H Carlsten H. Estrogens in rheumatoid arthritis; the immune system and bone. *Mol Cell Endocrinol.* (2011) 335:14–29. 10.1016/j.mce.2010.05.018 20685609

[20] Zaman G Jessop H Muzylak M De Souza R Pitsillides A Price J et al. Osteocytes use estrogen receptor alpha to respond to strain but their ERalpha content is regulated by estrogen. *J Bone Miner Res.* (2006) 21:1297–306. 10.1359/jbmr.060504 16869728

[21] Recker R Davies K Dowd R Heaney R. The effect of low-dose continuous estrogen and progesterone therapy with calcium and vitamin d on bone in elderly women. A randomized, controlled trial. *Ann Intern Med.* (1999) 130:897–904. 10.7326/0003-4819-130-11-199906010-00005 10375338

[22] D’Elia H Larsen A Mattsson L Waltbrand E Kvist G Mellstrom D et al. Influence of hormone replacement therapy on disease progression and bone mineral density in rheumatoid arthritis. *J Rheumatol.* (2003) 30:1456–63. 10.1186/isrctn46523456 12858441

[23] Bruce B Fries J. The health assessment questionnaire (HAQ). *Clin Exp Rheumatol.* (2005) 23:S14–8. 10.1002/acr.20620 16273780

[24] Tu X Delgado-Calle J Condon K Maycas M Zhang H Carlesso N et al. Osteocytes mediate the anabolic actions of canonical wnt/beta-catenin signaling in bone. *Proc Natl Acad Sci U S A.* (2015) 112:E478–86. 10.1073/pnas.1409857112 25605937 PMC4321271

[25] Smith E Gilligan C. Physical activity effects on bone metabolism. *Calcif Tissue Int.* (1991) 49:S50–4. 10.1007/BF02555089 1933599

[26] Furuya T. Clinical observations of osteoporosis in japanese patients with rheumatoid arthritis. *Mod Rheumatol.* (2022) 32:839–45. 10.1093/mr/roab130 34979563

[27] Williams C Anastasopoulou C Sapra A. *Biochemical Markers of Osteoporosis.* Treasure Island, FL: StatPearls (2025). 32644732

[28] Tariq S Tariq S Lone K Khaliq S. Alkaline phosphatase is a predictor of bone mineral density in postmenopausal females. *Pak J Med Sci.* (2019) 35:749–53. 10.12669/pjms.35.3.188 31258588 PMC6572960

[29] Chen R Gong K Chen W Chen Z Hua X Tan J et al. Association of serum alkaline phosphatase levels with bone mineral density, osteoporosis prevalence, and mortality in US adults with osteoporosis: evidence from NHANES 2005-2018. *Osteoporos Int.* (2025) 36:283–97. 10.1007/s00198-024-07324-w 39611944

[30] Tan L Long T Guan X Wu S Zheng W Fu H et al. Diagnostic value of vitamin d status and bone turnover markers in rheumatoid arthritis complicated by osteoporosis. *Ann Clin Lab Sci.* (2018) 48:197–204. 10.26226/morressier.5a79cbadd462b8029238baa5 29678847

[31] Bhattoa H Vasikaran S Trifonidi I Kapoula G Lombardi G Jorgensen N et al. Update on the role of bone turnover markers in the diagnosis and management of osteoporosis: a consensus paper from the european society for clinical and economic aspects of osteoporosis, osteoarthritis and musculoskeletal diseases (ESCEO), international osteoporosis foundation (IOF), and international federation of clinical chemistry and laboratory medicine (IFCC). *Osteoporos Int.* (2025) 36:579–608. 10.1007/s00198-025-07422-3 40152990 PMC12064614

[32] Yamaguchi T Sugimoto T Yano S Yamauchi M Sowa H Chen Q et al. Plasma lipids and osteoporosis in postmenopausal women. *Endocr J.* (2002) 49:211–7. 10.1507/endocrj.49.211 12081241

[33] Jeong I Cho S Kim S Choi H Park K Kim S et al. Lipid profiles and bone mineral density in pre- and postmenopausal women in Korea. *Calcif Tissue Int.* (2010) 87:507–12. 10.1007/s00223-010-9427-3 20976443

[34] Li S Guo H Liu Y Wu F Zhang H Zhang Z et al. Relationships of serum lipid profiles and bone mineral density in postmenopausal Chinese women. *Clin Endocrinol.* (2015) 82:53–8. 10.1111/cen.12616 25279969

[35] Chen H Shao Z Gao Y Yu X Huang S Zeng P. Are blood lipids risk factors for fracture? Integrative evidence from instrumental variable causal inference and mediation analysis using genetic data. *Bone.* (2020) 131:115174. 10.1016/j.bone.2019.115174 31785374

[36] Wu Q Gu S Chen Y He Y Xue M Guo F et al. Relationship between serum ApoB-100 and lumbar bone mineral density in postmenopausal women: a retrospective analysis of a health screening population. *Front Endocrinol.* (2025) 16:1667161. 10.3389/fendo.2025.1667161 41113705 PMC12527847

[37] He Y Liu Y Li R Xiang A Chen X Yu Q et al. The role of autophagy/lipophagy in the response of osteoblastic cells to hyperlipidemia (Review). *Exp Ther Med.* (2024) 28:328. 10.3892/etm.2024.12617 38979020 PMC11229398

[38] Singh L Tyagi S Myers D Duque G. Good, bad, or ugly: the biological roles of bone marrow fat. *Curr Osteoporos Rep.* (2018) 16:130–7. 10.1007/s11914-018-0427-y 29476394

[39] Fan L Chen J Chen C Zhang Y Yang Y Chen Z. The diagnostic value of the combined application of blood lipid metabolism markers and interleukin-6 in osteoporosis and osteopenia. *Lipids Health Dis.* (2025) 24:38. 10.1186/s12944-025-02456-2 39910539 PMC11796166

[40] Hauser B Riches P Wilson J Horne A Ralston S. Prevalence and clinical prediction of osteoporosis in a contemporary cohort of patients with rheumatoid arthritis. *Rheumatology.* (2014) 53:1759–66. 10.1093/rheumatology/keu162 24764264

[41] Chu Y Xu Y Ma L Wang J Zong H Tong W et al. Skeletal muscle index together with body mass index is associated with secondary osteoporosis in patients with rheumatoid arthritis. *Eur J Med Res.* (2024) 29:61. 10.1186/s40001-024-01665-2 38245751 PMC10799370

[42] Baker J England B George M Wysham K Johnson T Kunkel G et al. Elevations in adipocytokines and mortality in rheumatoid arthritis. *Rheumatology.* (2022) 61:4924–34. 10.1093/rheumatology/keac191 35325041 PMC9707328

