# Supplementary material for: Development and validation of a nomogram predicting osteoporosis risk in rheumatoid arthritis
Source: Front Med (Lausanne). 2026 Apr 29;13:1747090. doi: 10.3389/fmed.2026.1747090 (PMC13167499; doi:10.3389/fmed.2026.1747090)
Supplement: Supplementary file 1 [file Table_1.docx]

**Supplementary Table S1. **Missing Data Summary for Candidate Variables****

| **Variable** | **Missing Values (n)** | **Missing Percentage (%)** | **Handling Method Applied** |
| --- | --- | --- | --- |
| Gender | 0 | 0.0 | Complete-case analysis |
| Whether to use NSAIDs | 2 | 0.6 | Complete-case analysis |
| Whether to use hormone drugs | 5 | 1.4 | Complete-case analysis |
| Whether to use immune inhibitors | 3 | 0.9 | Complete-case analysis |
| Whether to use biological agents | 1 | 0.3 | Complete-case analysis |
| AKA | 18 | 5.2 | Multiple imputation |
| ANA | 14 | 4.0 | Complete-case analysis |
| SSA60KD | 22 | 6.3 | Multiple imputation |
| SSA52KD | 21 | 6.0 | Multiple imputation |
| SSB | 19 | 5.4 | Multiple imputation |
| ILD | 7 | 2.0 | Complete-case analysis |
| Hypertension | 0 | 0.0 | Complete-case analysis |
| Diabetes | 0 | 0.0 | Complete-case analysis |
| History of smoking | 3 | 0.9 | Complete-case analysis |
| Age | 0 | 0.0 | Complete-case analysis |
| Course of disease (months) | 6 | 1.7 | Complete-case analysis |
| TJC | 4 | 1.1 | Complete-case analysis |
| SJC | 4 | 1.1 | Complete-case analysis |
| VAS | 9 | 2.6 | Complete-case analysis |
| PaGA | 11 | 3.2 | Complete-case analysis |
| MDGA | 12 | 3.4 | Complete-case analysis |
| DAS28 | 8 | 2.3 | Complete-case analysis |
| CDAI | 15 | 4.3 | Complete-case analysis |
| SDAI | 17 | 4.9 | Complete-case analysis |
| HAQ-DI | 7 | 2.0 | Complete-case analysis |
| ESR | 3 | 0.9 | Complete-case analysis |
| CRP | 4 | 1.1 | Complete-case analysis |
| RF | 6 | 1.7 | Complete-case analysis |
| WBC | 2 | 0.6 | Complete-case analysis |
| Monocyte Count | 5 | 1.4 | Complete-case analysis |
| RBC | 2 | 0.6 | Complete-case analysis |
| Hb | 2 | 0.6 | Complete-case analysis |
| MPV | 10 | 2.9 | Complete-case analysis |
| NLR | 8 | 2.3 | Complete-case analysis |
| PLR | 8 | 2.3 | Complete-case analysis |
| MLR | 8 | 2.3 | Complete-case analysis |
| AST | 3 | 0.9 | Complete-case analysis |
| ALT | 3 | 0.9 | Complete-case analysis |
| Alb | 5 | 1.4 | Complete-case analysis |
| Alkaline Phosphatase | 4 | 1.1 | Complete-case analysis |
| LDH | 12 | 3.4 | Complete-case analysis |
| CK | 13 | 3.7 | Complete-case analysis |
| BUN | 3 | 0.9 | Complete-case analysis |
| Cr | 3 | 0.9 | Complete-case analysis |
| UA | 4 | 1.1 | Complete-case analysis |
| Ca | 6 | 1.7 | Complete-case analysis |
| P | 7 | 2.0 | Complete-case analysis |
| TC | 5 | 1.4 | Complete-case analysis |
| TG | 5 | 1.4 | Complete-case analysis |
| HDLC | 6 | 1.7 | Complete-case analysis |
| LDLC | 7 | 2.0 | Complete-case analysis |
| ApoA1 | 9 | 2.6 | Complete-case analysis |
| ApoB | 9 | 2.6 | Complete-case analysis |
| ApoA1/ApoB | 9 | 2.6 | Complete-case analysis |
| Lpa | 26 | 7.4 | Multiple imputation |
| FFA | 16 | 4.6 | Complete-case analysis |
| BMI | 2 | 0.6 | Complete-case analysis |

*Note: Total N = 349. Variables with <5% missing were handled by complete-case analysis; variables with 5–20% missing were handled by multiple imputation. No candidate variable in this table exceeded the 20% missing threshold for exclusion.*
