# Supplementary material for: Development and validation of a nomogram predicting osteoporosis risk in rheumatoid arthritis
Source: Front Med (Lausanne). 2026 Apr 29;13:1747090. doi: 10.3389/fmed.2026.1747090 (PMC13167499; doi:10.3389/fmed.2026.1747090)
Supplement: Supplementary file 2 [file Table_2.docx]

Table S2 Comparison of baseline clinical and demographic characteristics between the training and validation cohorts

|  | Overall | Training cohort | Validation cohort | p |
| --- | --- | --- | --- | --- |
| Variables | n=349 | n=250 | n=99 |  |
| Osteoporosis, n (%) |  |  |  | 0.615 |
| No | 217 (62.18) | 158 (63.20) | 59 (59.60) |  |
| Yes | 132 (37.82) | 92 (36.80) | 40 (40.40) |  |
| Gender, n (%) |  |  |  | 0.524 |
| Male | 99 (28.37) | 68 (27.20) | 31 (31.31) |  |
| Female | 250 (71.63) | 182 (72.80) | 68 (68.69) |  |
| Whether to use NSAIDs, n (%) |  |  |  | 0.647 |
| No | 34 (9.74) | 26 (10.40) | 8 (8.08) |  |
| Yes | 315 (90.26) | 224 (89.60) | 91 (91.92) |  |
| Whether to use hormone drugs, n (%) |  |  |  | 0.905 |
| No | 215 (61.60) | 155 (62.00) | 60 (60.61) |  |
| Yes | 134 (38.40) | 95 (38.00) | 39 (39.39) |  |
| Whether to use immune inhibitors, n (%) |  |  |  | 0.186 |
| No | 155 (44.41) | 105 (42.00) | 50 (50.51) |  |
| Yes | 194 (55.59) | 145 (58.00) | 49 (49.49) |  |
| Whether to use biological agents, n (%) |  |  |  | 1 |
| No | 313 (89.68) | 224 (89.60) | 89 (89.90) |  |
| Yes | 36 (10.32) | 26 (10.40) | 10 (10.10) |  |
| AKA, n (%) |  |  |  | 0.907 |
| Negative | 127 (36.39) | 90 (36.00) | 37 (37.37) |  |
| Positive | 222 (63.61) | 160 (64.00) | 62 (62.63) |  |
| ANA, n (%) |  |  |  | 0.256 |
| Negative | 281 (80.52) | 197 (78.80) | 84 (84.85) |  |
| Positive | 68 (19.48) | 53 (21.20) | 15 (15.15) |  |
| SSA60KD, n (%) |  |  |  | 0.885 |
| Negative | 325 (93.12) | 232 (92.80) | 93 (93.94) |  |
| Positive | 24 (6.88) | 18 (7.20) | 6 (6.06) |  |
| SSA52KD, n (%) |  |  |  | 0.516 |
| Negative | 317 (90.83) | 225 (90.00) | 92 (92.93) |  |
| Positive | 32 (9.17) | 25 (10.00) | 7 (7.07) |  |
| SSB, n (%) |  |  |  | 0.075 |
| Negative | 338 (96.85) | 239 (95.60) | 99 (100.00) |  |
| Positive | 11 (3.15) | 11 (4.40) | 0 (0.00) |  |
| ILD, n (%) |  |  |  | 0.955 |
| Negative | 316 (90.54) | 227 (90.80) | 89 (89.90) |  |
| Positive | 33 (9.46) | 23 (9.20) | 10 (10.10) |  |
| Hypertension, n (%) |  |  |  | 1 |
| No | 241 (69.05) | 173 (69.20) | 68 (68.69) |  |
| Yes | 108 (30.95) | 77 (30.80) | 31 (31.31) |  |
| Diabetes, n (%) |  |  |  | 1 |
| No | 308 (88.25) | 221 (88.40) | 87 (87.88) |  |
| Yes | 41 (11.75) | 29 (11.60) | 12 (12.12) |  |
| History of smoking, n (%) |  |  |  | 0.822 |
| No | 262 (75.07) | 189 (75.60) | 73 (73.74) |  |
| Yes | 87 (24.93) | 61 (24.40) | 26 (26.26) |  |
| Age (median [IQR]) | 62.000 [56.000, 69.000] | 61.000 [56.000, 68.750] | 64.000 [56.500, 71.000] | 0.189 |
| Course of disease (months) (median [IQR]) | 48.000 [6.000, 120.000] | 48.000 [6.000, 120.000] | 36.000 [6.000, 120.000] | 0.273 |
| TJC (median [IQR]) | 7.000 [4.000, 12.000] | 7.000 [4.000, 12.000] | 7.000 [4.000, 11.000] | 0.893 |
| SJC (median [IQR]) | 3.000 [1.000, 5.000] | 3.000 [1.000, 6.000] | 2.000 [1.000, 5.000] | 0.721 |
| VAS (median [IQR]) | 5.000 [3.000, 6.000] | 5.000 [3.125, 6.000] | 5.000 [3.000, 6.000] | 0.785 |
| PaGA (median [IQR]) | 50.000 [30.000, 60.000] | 50.000 [31.250, 60.000] | 50.000 [30.000, 60.000] | 0.785 |
| MDGA (median [IQR]) | 40.000 [30.000, 60.000] | 40.000 [30.000, 60.000] | 40.000 [30.000, 60.000] | 0.926 |
| DAS28 (median [IQR]) | 4.650 [4.019, 5.328] | 4.686 [4.003, 5.402] | 4.596 [4.071, 5.163] | 0.420 |
| CDAI (median [IQR]) | 19.000 [13.000, 27.000] | 19.000 [13.000, 28.000] | 20.000 [13.500, 26.000] | 0.869 |
| SDAI (median [IQR]) | 38.500 [24.600, 71.200] | 40.320 [24.230, 72.800] | 33.960 [24.805, 64.300] | 0.424 |
| HAQ-DI (median [IQR]) | 0.750 [0.450, 1.200] | 0.750 [0.450, 1.150] | 0.750 [0.450, 1.250] | 0.555 |
| ESR (median [IQR]) | 50.000 [28.000, 74.000] | 50.000 [29.000, 74.000] | 48.000 [26.000, 67.500] | 0.295 |
| CRP (median [IQR]) | 15.400 [5.530, 50.300] | 16.050 [5.742, 50.975] | 12.800 [5.060, 39.100] | 0.365 |
| RF (median [IQR]) | 143.000 [34.600, 492.000] | 131.500 [39.475, 486.500] | 190.000 [23.550, 507.000] | 0.912 |
| WBC (median [IQR]) | 6.700 [5.100, 8.300] | 6.700 [5.100, 8.400] | 6.700 [5.100, 7.950] | 0.544 |
| Monocyte Count, (median [IQR]) | 0.460 [0.350, 0.610] | 0.460 [0.340, 0.617] | 0.470 [0.360, 0.590] | 0.982 |
| RBC (median [IQR]) | 3.920 [3.560, 4.210] | 3.905 [3.552, 4.207] | 3.960 [3.570, 4.205] | 0.843 |
| Hb (median [IQR]) | 114.000 [102.000, 125.000] | 114.000 [100.250, 124.750] | 116.000 [106.000, 124.500] | 0.550 |
| MPV (median [IQR]) | 10.300 [9.500, 11.200] | 10.250 [9.500, 11.075] | 10.400 [9.800, 11.500] | 0.148 |
| NLR (median [IQR]) | 2.941 [2.040, 4.390] | 3.087 [2.118, 4.607] | 2.664 [1.990, 3.820] | 0.054 |
| PLR (median [IQR]) | 173.762 [126.291, 250.000] | 178.736 [129.743, 258.977] | 162.774 [115.009, 237.933] | 0.083 |
| MLR (median [IQR]) | 0.321 [0.226, 0.420] | 0.331 [0.227, 0.434] | 0.309 [0.228, 0.393] | 0.199 |
| AST (median [IQR]) | 19.000 [15.000, 24.000] | 19.000 [15.000, 25.000] | 18.000 [15.000, 24.000] | 0.667 |
| ALT (median [IQR]) | 14.000 [10.000, 21.000] | 15.000 [10.000, 21.000] | 14.000 [10.500, 21.000] | 0.920 |
| Alb (median [IQR]) | 34.600 [31.800, 37.500] | 34.500 [31.825, 37.400] | 34.900 [31.750, 37.900] | 0.461 |
| Alkaline Phosphatase (median [IQR]) | 90.000 [73.000, 107.000] | 90.000 [73.000, 106.000] | 83.000 [73.500, 113.500] | 0.579 |
| LDH (median [IQR]) | 209.000 [178.000, 248.000] | 210.000 [176.500, 249.750] | 204.000 [179.000, 235.500] | 0.345 |
| CK (median [IQR]) | 37.000 [25.000, 54.000] | 37.000 [26.000, 54.000] | 40.000 [25.000, 56.000] | 0.485 |
| BUN (median [IQR]) | 5.700 [4.500, 7.100] | 5.600 [4.500, 7.100] | 6.100 [4.700, 7.200] | 0.228 |
| Cr (median [IQR]) | 53.000 [46.000, 63.000] | 52.000 [45.000, 62.000] | 55.000 [47.500, 64.500] | 0.059 |
| UA (median [IQR]) | 284.000 [234.000, 347.000] | 290.000 [230.000, 348.750] | 279.000 [243.500, 342.000] | 0.783 |
| Ca (median [IQR]) | 2.200 [2.140, 2.270] | 2.200 [2.140, 2.270] | 2.210 [2.160, 2.265] | 0.520 |
| P (median [IQR]) | 1.210 [1.080, 1.330] | 1.210 [1.080, 1.330] | 1.220 [1.090, 1.340] | 0.758 |
| TC (median [IQR]) | 4.300 [3.700, 5.100] | 4.250 [3.700, 5.000] | 4.600 [3.750, 5.300] | 0.129 |
| TG (median [IQR]) | 1.090 [0.820, 1.490] | 1.070 [0.812, 1.490] | 1.160 [0.875, 1.570] | 0.103 |
| HDLC (median [IQR]) | 1.120 [0.930, 1.310] | 1.105 [0.930, 1.290] | 1.140 [0.930, 1.330] | 0.317 |
| LDLC (median [IQR]) | 2.860 [2.370, 3.370] | 2.805 [2.370, 3.350] | 2.930 [2.415, 3.440] | 0.159 |
| ApoA1 (median [IQR]) | 1.270 [1.090, 1.430] | 1.270 [1.082, 1.430] | 1.280 [1.095, 1.460] | 0.654 |
| ApoB (median [IQR]) | 0.760 [0.620, 0.920] | 0.750 [0.613, 0.890] | 0.790 [0.645, 0.960] | 0.088 |
| ApoA1/ApoB (median [IQR]) | 1.700 [1.300, 2.100] | 1.700 [1.300, 2.100] | 1.700 [1.300, 2.000] | 0.903 |
| Lpa (median [IQR]) | 203.500 [98.700, 400.000] | 212.900 [101.450, 418.725] | 169.500 [80.750, 376.100] | 0.105 |
| FFA (median [IQR]) | 0.350 [0.220, 0.480] | 0.340 [0.220, 0.480] | 0.360 [0.225, 0.460] | 0.812 |
| BMI (median [IQR]) | 22.600 [20.703, 25.110] | 22.710 [20.718, 25.337] | 22.531 [20.658, 24.876] | 0.810 |
